# Supplementary material for: Pediatric Polytrauma Fire Victim Simulation
Source: MedEdPORTAL. 2024 Feb 27;20:11383. doi: 10.15766/mep_2374-8265.11383 (PMC10897059; doi:10.15766/mep_2374-8265.11383)
Supplement: Supplementary file 1 — Polytrauma Fire Sim Case.docxSim Environment Checklist.docxEKG, CXR, FAST, and Labs.docxPolytrauma Fire Debriefing Guide.docxPolytrauma Fire Victim Sim Survey.docxPolytrauma Debriefing.pptxPolytrauma Reference Sheet.docx [file mep_2374-8265.11383-s001.zip › C. EKG, CXR, FAST, and Labs.docx]

**Appendix C:** EKG, CXR, FAST, and Labs

Instructions: These images and tables should be printed out in advance of the simulation to be handed out to the team leader as they are “ordered” during the care of the patient.

EKG
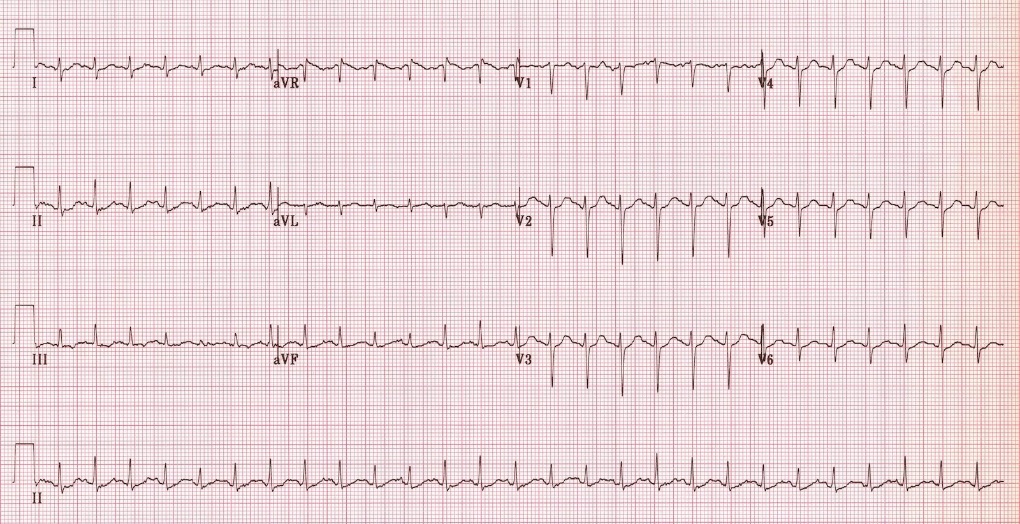


Author Owned

Chest X-Ray

Post Intubation

**
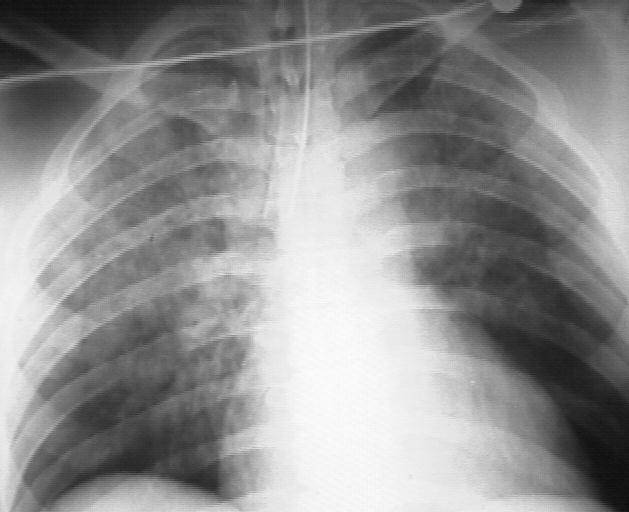
**

Author Owned

Suprapubic FAST


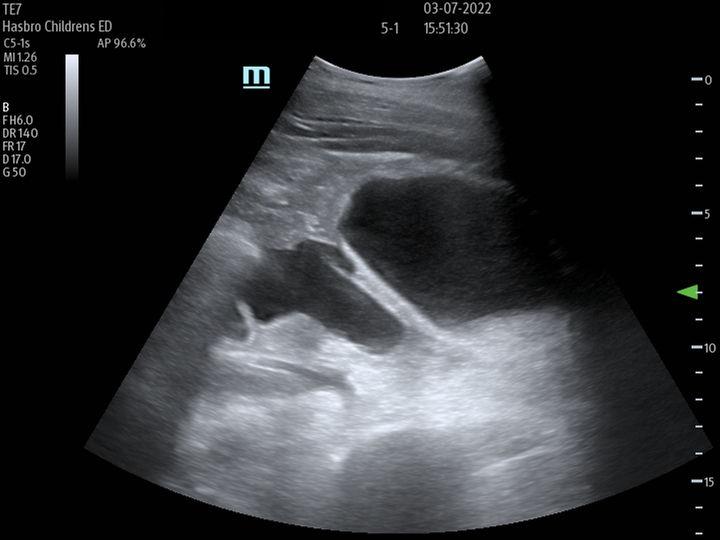


Author Owned

Lab results

**VBG**:

| **pH** | 7.10 | Ref (7.35-7.45) |
| --- | --- | --- |
| **pCO_2_** | 53 mmHg | Ref (35-45) |
| **pO_2_** | 60 mmHg | Ref (19-65) |
| **HCO_3_** | 6 mmol/L | Ref (23-29) |
| **BE** | -16 mmol/L | Ref(-2-3) |
| **Lactate** | 15 mmol/L | Ref (0-1) |

| **Co Hgb** | 30% | Ref (0-3) |
| --- | --- | --- |
| **Met Hgb** | 1% | Ref (0) |

**BMP**:

| **Sodium** | 140 mmol/L | Ref (135-145) |
| --- | --- | --- |
| **Potassium** | 5.4 mmol/L | Ref (3.2-4.9) |
| **Chloride** | 104 mmol/L | Ref (95-110) |
| **CO_2_** | 6 mmol/L | Ref (21-23) |
| **BUN** | 15 mmol/L | Ref (7-18) |
| **Creatinine** | 0.4 mmol/L | Ref (0.6-1.3) |
| **Glucose** | 150 mg/dL | Ref (70-100) |

**CBC**:

| **WBC** | 14.0/nL | Ref (5.5-15.5) |
| --- | --- | --- |
| **HGB** | 16 g/dL | Ref (12.5-15.0) |
| **HCT** | 45% | Ref (37-45) |
| **PLT** | 190/nL | Ref (150-450) |
