# Supplementary material for: Pediatric Polytrauma Fire Victim Simulation
Source: MedEdPORTAL. 2024 Feb 27;20:11383. doi: 10.15766/mep_2374-8265.11383 (PMC10897059; doi:10.15766/mep_2374-8265.11383)
Supplement: Supplementary file 1 — Polytrauma Fire Sim Case.docxSim Environment Checklist.docxEKG, CXR, FAST, and Labs.docxPolytrauma Fire Debriefing Guide.docxPolytrauma Fire Victim Sim Survey.docxPolytrauma Debriefing.pptxPolytrauma Reference Sheet.docx [file mep_2374-8265.11383-s001.zip › D. Polytrauma Fire Debriefing Guide.docx]

**Appendix D:** Polytrauma Fire Victim Simulation Debriefing Guide

**Estimated Duration: 30 minutes**

This debriefing guide is intended to be used for the preparation of facilitators to ensure standardized teaching. It serves as a guide during the debriefing process for focus points to guide time management. This simulation can be debriefed in whichever style best suits your institution. However, for the purposes of this research, the debrief was conducted as a mix of scripted debriefing as well as Advocacy and Inquiry (AI).^1^ Scripted debriefing was essential for time management, while AI allowed for deeper reflective discussion into management decisions that were incorrect or varied from the expected progression. In the AI framework, the fundamental principle is reflective discussion based in genuine curiosity in which the facilitator seeks to understand the mental framework of participants that led to certain decision making, whether positive or negative.

Our debriefs started with an introduction to the debriefing phase in order to mark the transition from the active phase to the reflective phase. In the introduction, ground rules were set including the expectation that all participants (facilitators and learners) are intelligent, acting in good faith and to the best of their abilities. The debrief itself moves through several phases. The first phase is an opportunity to defuse emotional tension lingering from the case participation before delving into a reflection on learning points. Also, the facilitator or team leader provides a concise SBAR (Situation Background Assessment Recommendation) summary of the clinical scenario to ensure that all participants share a mental model for the discussion to come. Next, we discuss the communication and teamwork portions of the case. Last, we cover the medical management.

| **Debriefing Guide** | | |
| --- | --- | --- |
| **Debriefing Phase** | **Suggested questions/phrases** | **Case specific discussion points and references** |
| **Emotional Debrief/ Case Summary** | - *“This was a difficult case. Can each team member give a one word on how they’re feeling emotionally after this case?* - *“Would the team leader please summarize the key elements of the case?”* | Validate the feelings of learners, but do not perseverate on this part- it should not cut into medical management discussion.  This was a 6-year-old boy brought in by EMS after extraction from a car accident. He was entrapped in the vehicle and exposed to smoke and fire. His injuries included an unstable airway, partial thickness circumferential burns, hemoperitoneum, and metabolic derangements consistent with carbon monoxide and cyanide toxicity. |
|  |  | |

| **Teamwork and Communication** | - *“Was there a clearly identified team leader?”* - *“Were roles assigned and did everyone feel like they knew their roles?”* - Comment on any other specific instances of successes or opportunities for improvement in communication and teamwork | **Team Leader –** identifies self, commands the room but also invites thoughts from the entire team. Stands at the foot of the bed.  **Airway physician** – Communicates with team leader about changes in airway status, coordinates timing of airway interventions, requests RT support as needed  **Exam/Survey physician** – Completes thorough and organized primary and secondary survey and communicates findings loudly and clearly to the entire team |
| --- | --- | --- |
|  |  | |

| **Medical Management: Pediatric Airway Considerations** | - *“What was your assessment of this patient’s airway?”* - *“I noticed you chose ____ for your airway, how did you come to that decision?”* - *“I noticed that you used direct laryngoscopy for this patient. Inhalational burns can cause airway edema, and I think video laryngoscopy would be a better approach. What was your rationale for your approach?”* - *“I saw you call for succinylcholine as your paralytic. Generally, rocuronium is preferred in pediatric patients and patients at risk for hyperkalemia. What was your rationale for choosing succinylcholine?”* - *“This was a critical airway, what special considerations did you make in creating your airway plan?”* | Endotracheal Tube (ETT) Sizing   - Age/4 + 3.5 (cuffed – always use cuffed if available) - Be prepared with smaller size if risk of airway edema - 1 x ETT = (age/4) + 3.5 [Cuffed] - 2 x ETT = Naso- or orogastric tube or foley size - 3 x ETT = ETT depth - 4 x ETT = maximum chest tube size   Rapid Sequence Intubation   - Rocuronium is preferred over succinylcholine for intubation of pediatric patients   Backup airway plans:   - Call for help early! - Video Laryngoscopy, bougie, fiberoptic - Cricothyrotomy: open if above 12 years old; Needle cricothyrotomy if younger than 12 years old - Retrograde (reverse) intubation |
| --- | --- | --- |
|  |  | |
| **Medical Management: Pediatric Trauma and Burns** | - *“I noticed that you ordered 1L of saline as initial resuscitation of this patient. Standard pediatric bolus dosing starts with 20cc/kg. I wonder what your rationale was in choosing that volume?”* - *As you completed the secondary survey you did/did not identify that the patient had hemoperitoneum. How did you approach the management of this?”* - *I saw you ordered uncrossmatched blood for this patient. His systolic blood pressure remained above the hypotensive limit for age. I wonder what your concern was in ordering the blood?”* - *“This patient had a compensated blood pressure; how do you determine the appropriate minimum blood pressure in children?”* | - ATLS protocol of ABCDE primary and secondary surveys - Exposure is where you will identify burn patterns including circumferential burns - %BSA = body surface percentage of partial/full thickness burns only - Palmar surface area = 0.5-2% depending on BMI^2^ - Assess for circumferential burns which will require escharotomy - Fluid Resuscitation^3^:   - If > 10% TBSA (children): 3 mL/kg/%burn   - ½ in first 8 hours, ½ in next 16 hours   - Titrate hourly rate to urine output   - Target urine output is 1-2 mL/kg/hr   - Include maintenance fluids - Normal vitals in children   - Reference PALS cards or phone apps   - Minimum systolic BP = 70 + (age*2) - CPR in children   - Rate: 100-120 BPM   - 15:2 with 2 people   - Continuous with advanced airway |
|  |  | |
| **Medical Management: Cyanide Poisoning^4^** | - *“I noticed you obtained a blood gas on this patient, what was your interpretation of the metabolic derangement?”* - *“You did/did not identify cyanide poisoning in this patient. What about this patient’s presentation put them at risk for a cyanide exposure? What are some considerations in choosing the treatment modality?”* | - Empiric CN poisoning treatment in a metabolic acidosis with high lactate in this clinical scenario - Caused by victim entrapment in a fire where synthetic materials are combusting - Lab findings: anion gap metabolic acidosis, high lactate, high venous O2 sat - Caused by: Electron transport chain uncoupling 🡪 metabolic (lactic) acidosis - Treatment:   - Hydroxocobalamin (Cyanokit) – binds CN to form B12. First line, but bright red and will affect lab work, so draw off blood for labs before starting treatment   - Secondary treatment: Na Thiosulfate.   - Avoid Amyl and Na Nitrite- Converts Hgb to methemoglobin; CN preferentially binds methemoglobin. But this causes methemoglobinemia & hypotension.   - Do not delay treatment by waiting for lab results. Treat empirically if clinical evidence of CN poisoning. |
|  |  | |
| **Medical Management: Carbon Monoxide Poisoning^5^** | - *“You did/did not identify carbon monoxide poisoning in this patient. What about this patient’s presentation puts them at risk for CO poisoning?”* - *“What are the options for treatment in this patient with so many competing medical needs?”* | - Range of Abnormal: 5%-60% - Caused by: Displaces O_2_ from Hgb (CO has 200x greater binding affinity for Hgb than O_2_) – therefore may cause falsely elevated SpO2 readings on pulse oximetry. - Symptoms:   - 10-30%: Headache   - 30-40%: Severe headache, weakness, nausea, dizziness   - 40-60%: tachycardia, tachypnea, seizures, loss of consciousness   - > 60%: death - Treatment: Pure O_2_ + hyperbarics - Half-life of CO = 300 min (room air), 90min (nonrebreather), 30min (hyperbaric) - Hyperbaric if persistent metabolic acidosis, altered mental status, myocardial infarction, CO >25% (10% if pregnant) |
|  |  | |
| **Medical management: Triage** | - *“This case had many components. How do you approach the medical triage of the care of a patient with multiple simultaneous needs?”* | - Highlight the importance of the ATLS (Advanced Trauma Life Support) algorithm, and stabilization of each component |

1. Rudolph JW, Simon R, Dufresne RL, Raemer DB. There’s no such thing as “nonjudgmental” debriefing: a theory and method for debriefing with good judgment. Simul Healthc. 2006;1(1):49-55. doi:10.1097/01266021-200600110-00006
2. Pham C, Collier Z, Gillenwater J. Changing the Way We Think About Burn Size Estimation. J Burn Care Res. 2019;40(1):1-11. doi:10.1093/jbcr/iry050
3. Mehta M, Tudor GJ. Parkland Formula. In: StatPearls. StatPearls Publishing; 2022. Accessed August 2, 2022. <http://www.ncbi.nlm.nih.gov/books/NBK537190/>
4. Cyanide poisoning - UpToDate. Accessed August 2, 2022. <https://www.uptodate.com/contents/cyanide-poisoning?search=cyanide%20poisoning&source=search_result&selectedTitle=1~53&usage_type=default&display_rank=1>
5. Carbon monoxide poisoning - UpToDate. Accessed August 2, 2022. <https://www.uptodate.com/contents/carbon-monoxide-poisoning?search=carbon%20monoxide%20poisoning&source=search_result&selectedTitle=1~96&usage_type=default&display_rank=1>
