# Supplementary material for: Pediatric Polytrauma Fire Victim Simulation
Source: MedEdPORTAL. 2024 Feb 27;20:11383. doi: 10.15766/mep_2374-8265.11383 (PMC10897059; doi:10.15766/mep_2374-8265.11383)
Supplement: Supplementary file 1 — Polytrauma Fire Sim Case.docxSim Environment Checklist.docxEKG, CXR, FAST, and Labs.docxPolytrauma Fire Debriefing Guide.docxPolytrauma Fire Victim Sim Survey.docxPolytrauma Debriefing.pptxPolytrauma Reference Sheet.docx [file mep_2374-8265.11383-s001.zip › E. Polytrauma Fire Victim Sim Survey.docx]

**Appendix E:** Polytrauma Fire Victim Simulation Case Survey

Instructions – This may be used following the case and debrief to collect feedback from participants on the case

1. What is your current level of training?

☐ PGY 1 ☐ PGY 2 ☐ PGY 3 ☐ PGY 4

☐ PGY 5 ☐ PGY 6 ☐ PGY 7 ☐ Attending Physician

☐ Other _______________________

1. If you are a resident, what residency program are you in?

☐ Emergency Medicine ☐ Pediatrics ☐ Other _______________________

1. Approximately, how many patients with inhalation injury have you cared for in your medical career? ______
2. Approximately, how many patients with CO toxicity have you cared for in your medical career? ______
3. Approximately, how many patients with CN toxicity have you cared for in your medical career? ______

**Please rate your agreement with the following statements:**

|  |  | Strongly Disagree | Disagree | Neither Agree nor Disagree | Agree | Strongly Agree |
| --- | --- | --- | --- | --- | --- | --- |
| 6. | This simulation case provided is relative to my work. | □ | □ | □ | □ | □ |
| 7. | This simulation case was realistic. | □ | □ | □ | □ | □ |
| 8. | This simulation case was effective in teaching trauma resuscitation skills. | □ | □ | □ | □ | □ |
| 9. | This simulation case was effective in teaching about evaluation and management of smoke inhalation injuries and metabolic derangements. | □ | □ | □ | □ | □ |
| 10. | The debrief created a safe environment. | □ | □ | □ | □ | □ |
| 11. | The debrief promoted reflection and team discussion. | □ | □ | □ | □ | □ |

**After participating in this session, how confident are you in your ability to:**

|  |  | Very Not confident | Not confident | Neutral | Confident | Very Confident |
| --- | --- | --- | --- | --- | --- | --- |
| 12 | Identify smoke inhalation injury and need for early intubation. | □ | □ | □ | □ | □ |
| 13 | Evaluate and manage CO toxicity. | □ | □ | □ | □ | □ |
| 14 | Evaluate and manage CN toxicity. | □ | □ | □ | □ | □ |
| 15 | Complete primary and secondary survey to avoid missing life-threatening injuries. | □ | □ | □ | □ | □ |

16. What did you take away from this case and/or how will it change your practice?

____________________________________________________________________

____________________________________________________________________

____________________________________________________________________

17. What specific changes would you make to improve this scenario?

____________________________________________________________________

____________________________________________________________________

18. Other comments or suggestions:

____________________________________________________________________

____________________________________________________________________

____________________________________________________________________

**Thank you for taking the time to complete this survey!**
