# Supplementary material for: Pediatric Polytrauma Fire Victim Simulation
Source: MedEdPORTAL. 2024 Feb 27;20:11383. doi: 10.15766/mep_2374-8265.11383 (PMC10897059; doi:10.15766/mep_2374-8265.11383)
Supplement: Supplementary file 1 — Polytrauma Fire Sim Case.docxSim Environment Checklist.docxEKG, CXR, FAST, and Labs.docxPolytrauma Fire Debriefing Guide.docxPolytrauma Fire Victim Sim Survey.docxPolytrauma Debriefing.pptxPolytrauma Reference Sheet.docx [file mep_2374-8265.11383-s001.zip › A. Polytrauma Fire Sim Case.docx]

| **SIMULATION CASE TITLE: Pediatric Polytrauma Fire Victim Simulation**  **Authors:** Lauren Vrablik, MD & Robyn Wing, MD, MPH  **Updated:** 10/8/20  **Learners:** Emergency Medicine Residents, Attendings, Advanced Practice Practitioners, Pediatric Emergency Medicine Fellows  **Physical setting:** (Pediatric) Emergency department  **Estimated duration:** 15 minutes for case + 30 min debrief | |
| --- | --- |
| **PATIENT NAME:** Michael Anton **PATIENT AGE:** 6  **CHIEF COMPLAINT:** Unresponsive following car accident | |
| **Brief narrative description of case** | A school van was in a collision with multiple casualties. EMS found the van on fire and extrication was prolonged. The patient was found trapped inside and has sustained burns and a large splenic laceration. |
| **Learning Objectives** | 1. Identify critical airway caused by smoke inhalation injury  2. Prepare for a difficult pediatric airway using video techniques  3. Manage Carbon Monoxide (CO) toxicity using oxygen  4. Identify Cyanide (CN) toxicity through situational exposure and metabolic derangements  5. Manage Cyanide (CN) toxicity using the cyanokit  6. Complete primary and secondary survey assessments to avoid missing injuries including hemoperitoneum and circumferential burns |
| **Critical Actions** | - Identify upper airway obstruction - Intubate early with alternative airway plan identified - Identify hemoperitoneum - Identify circumferential arm burns - Effectively communicate concerns of hemoperitoneum and burns to surgical team - Obtain lab work including Type and Screen, CN and CO levels - Initiate volume repletion - Initiate empiric treatment for CN poisoning - Stabilize and identify need for transfer to hyperbaric chamber |

**Appendix A:** Case - to be used by facilitators and techs during preparation and execution of the simulation

| Initial Presentation | | | | | |
| --- | --- | --- | --- | --- | --- |
| **Initial vital signs** | HR:130, RR: 32, BP: 86/60, O_2_ 88% on 10L NRB, Temp: 99 | | | | |
| **Overall Appearance** | Visibly burned 20% of body, soot around nares, minimally responsive | | | | |
| **Actors and roles in the room at case start** | **EMS:** You are appropriate and helpful, but appear stressed. Give brief overview of scene on arrival: Car accident was head on, there were multiple casualties. Extrication took 20 minutes due to active fire. Child was found trapped inside with lots of smoke. There was no resuscitation on scene – just a “scoop and run”. You placed a nonrebreather (NRB) immediately. He was initially awake/coughing, then developed stridor during transport and became obtunded. If asked - you did not obtain IV access given extremity burns. (EMS confederate can flex to be RT after conclusion of the report if staffing is limited)  **Nurse:** You are a trained trauma nurse and will perform all tasks requested. Do not offer suggestions of what to do. If being told to do multiple tasks at once, ask them to prioritize what should be done first. Remark about blood being “bright red” when you draw it.  **RT:** You assist with noninvasive ventilation if requested and ventilation after intubation.  **Facilitator**: Present either in person or in the control room for simulation equipment. This faculty instructor may provide feedback on exam findings (such as question about the degree of burns on the patient). The faculty instructor observes the performance of the learner(s), provides feedback and instruction to the nurse to facilitate case progression, and facilitates the debriefing session. | | | | |
| **HPI** | Michael is a 6 year-old male with no known PMH. He was in the school van with his class when there was a collision with fire. He was trapped and extrication took 20 minutes. He was awake but altered and coughing when picked up by EMS. Respiratory status worsened en route, and he developed stridor. He was placed on NRB. | | | | |
| **Past Medical/Surgical History** | **Medications** | | **Allergies** | | **Family/social History** |
| None Known | None Known | | None Known | | Parents not at bedside |
| **Physical Examination** | | | | | |
| General **– sleepy/altered**. Responsive to painful stim. Unable to answer questions. Groaning/coughing  Eyes - Normal  ENT – **Red lips, soot on face, erythema and swelling of oropharynx**.  Neck - Normal  Cardiovascular – Tachycardic, Regular, no m/r/g  Lungs - **+ inspiratory stridor, wheeze** throughout, tracheal tugging  Skin – **20% total body surface area mix of 2^nd^ and 3^rd^ degree burns** to chest, arms, head  Abdomen – **Distended, tender**, + guarding throughout, **bruise to upper abdomen**  Musculoskeletal – No obvious deformities, 2^nd^ and 3^rd^ degree burns to arms, **circumferential burns** to right arm  Neurological – Sleepy but arousable to painful stimuli. Unable to participate with exam. Noted to move all extremities in agitated fashion. | | | | | |
| Scenario Triggers and Progression | | | | | |
| **Patient State** | | **Trigger/Learner Action** | | **Progression** | |
| **State 1: Initial Presentation**  Rhythm: Sinus Tach  HR: 130/min  BP: 86/60  RR: 32/min  O_2_SAT: 88 % on NRB  T: 37.8^o^C | | 1. Ask for oxygen, monitors, and access | |  | |
|  |  | 1. Assess primary survey | |  | |
|  |  | 1. Oxygen via NRB/bag valve mask | | Saturation does not improve.  If not intubated within 5 minutes 🡪 State 4 (Respiratory  Arrest) | |
|  |  | 1. Identify need for intubation | | If succinylcholine used for RSI 🡪 State 5 (VTach Arrest)  Direct laryngoscopy intubation fails. Requires either video laryngoscopy or bougie. | |
|  |  | 1. Identify back up modality for intubation and have smaller endotracheal tube (ETT) size prepared | | Once intubated 🡪 State 2 (Sats come up to 95%) | |
| **State 2: Respiratory Stabilization**  Rhythm: Sinus Tach  HR: 110/min  BP: 84/60  RR: determined by bagging  O_2_SAT: 95 % intubated  T: 37.8^o^C | | 1. Obtain CXR for ETT placement | | Give CXR immediately after request (Appendix C) | |
|  |  | 1. Proceed with primary and secondary survey | | If team calls for surgery, facilitator states “Surgery is not available at this time; they’re at the bedside of a different crash victim. The surgical attending asks that you complete the full primary and secondary survey to help them triage severity.” | |
|  |  | 1. Identify seatbelt sign and hemoperitoneum | | Upon exposure of the abdomen or physical examination of the abdomen/pelvis facilitator should state “the abdomen is distended and rigid.”  If team does not request ultrasound for FAST, RN can prompt “An ultrasound is available if you need one.”  If team calls for surgery, facilitator states “Surgery is not available at this time; they’re at the bedside of a different crash victim. The surgical attending asks that you complete the full primary and secondary survey, call back to inform them of the findings, and optimize medically until they arrive.” | |
|  |  | 1. Identify circumferential burns (right arm) | | Escharotomy is not required for this case. If team asks about escharotomy, facilitator can state that the surgical team will manage it when they arrive. | |
|  |  | 1. Assess %BSA burned | | If not assessed, when team calls to report to “surgical attending” (fielded by the facilitator), the facilitator can say “The surgeon wants to know the total BSA of the burns.” | |
|  |  | 1. Start volume repletion (IV fluid or blood) | |  | |
|  |  | 1. Send labs including CBC, CMP, VBG, CO, CN, type and screen | | If team does not ask for blood work, RN can prompt “do you want any labs?” and/or facilitator can state “The surgeon wants to know if any labs are back yet for preop planning.”  When drawing blood the RN states “The blood is bright red!”  5 minutes after lab send or 5 minutes into State 2, labs result 🡪 State 3 (Stable Intubated) | |
| **State 3: Stable Intubated**  Rhythm: Sinus Tach  HR: 110/min  BP: 88/58  RR: determined by bagging  O_2_SAT: 95 % intubated  T: 37.8^o^C | | 1. Identify metabolic acidosis | | If not identified, nurse can state “is there anything we can do before he goes to the OR?” and/or facilitator can state “the surgical team requests you medically optimize him before the OR.”  If team reviews labs and does not identify metabolic derangements 🡪case end | |
|  |  | 1. Identify likely CN poisoning | | If not identified, nurse can state “is there anything we can do before he goes to the OR?” and/or facilitator can state “the surgical team requests you medically optimize him before the OR.”  If identified but team does not know how to manage, nurse can offer to call poison control.  If not completed, highlight in debrief.  If team reviews labs and does not identify metabolic derangements 🡪case end | |
|  |  | 1. Order hydroxocobalamin | | Once ordered 🡪 case end | |
|  |  | 1. Identify CO poisoning   Identify need to transfer for hyperbarics and operating room | | If not identified, nurse can state “is there anything we can do before he goes to the OR?” and/or facilitator can state “the surgical team requests you medically optimize him before the OR.”  If team does not know how to approach CO management, nurse can offer to call poison control.  If not completed, highlight in debrief.  Case End | |
| **State 4: Respiratory Arrest**  Rhythm: Sinus Tach  HR: 150/min  BP: 84/56  RR: determined by bagging  O_2_SAT: Slow trend down to 0%  T: 37.8^o^C | | 1. Troubleshoot BVM | | No improvement with noninvasive ventilation | |
|  |  | 1. Intubate with VL | | Once Intubated 🡪 State 2 | |
| **State 5: VTach Arrest**  Rhythm: VTach  HR: 0  BP: Unable to obtain  RR: 0  O_2_SAT: Slow trend down to 0%  T: 37.8^o^C | | 1. High Quality CPR (15:2) | |  | |
|  |  | 1. Administer hyperkalemia reversal agent or cardioprotective agent (fluids, CaGlu, or NaBicarb) | | If not administered, patient does not recover with defibrillation. If team does not treat the hyperkalemia, nurse can state “are there any reversible causes?” | |
|  |  | 1. Defibrillate x 1 | | Shock delivered after hyperkalemia treatment 🡪 State 6 (Post Arrest)  Shock delivered without hyperkalemia treatment 🡪 no response.  If team does not treat the hyperkalemia, nurse can state “are there any reversible causes?” | |
| **State 6: Post Arrest**  Rhythm: Sinus Tach  HR: 150/min  BP: 78/54  RR: determined by bagging  O_2_SAT: 95% with bagging  T: 37.8^o^C | | 1. Intubate if not already done | | Once intubated 🡪 State 2 | |
|  |  | 1. Volume repletion (IVF or blood) | |  | |
|  |  | 1. Vasopressor drip | |  | |

| Supporting Documents and media | |
| --- | --- |
| **Labs** | Na: 140, K:5.4, Cl: 104, HCO_3_: 6, BUN: 15, Cr: 0.4, Glu: 150, Lactate: 15 pH: 7.10, PCO_2_: 53. PO_2_: 60, HCO_3_: 6, WBC 14, HGB: 16, HCT: 45, Plt: 190 |
| **EKG** | Sinus Tachycardia |
| **Imaging** | CXRs: Post Intubation revealing inhalational injury and appropriately placed ETT  Ultrasound: positive FAST with free fluid in the abdomen |

**Ideal Scenario Flow:**

The team immediately assigns roles including team leader, airway, patient examiner, and bedside nurse, and request that the patient be placed on a monitor. Two points of large bore IV access are established - through burns if necessary. While the primary survey is underway, the team leader listens to the EMS transport story. At time of access, team leader requests a full panel of lab work including CBC, CMP, VBG with lactate, and Carbon Monoxide level. The provider performing the primary survey identifies that the airway is not intact due to soot around the nares and in the mouth with audible stridor, and announces the need for immediate intubation. The airway provider identifies the possibility of a difficult airway and need for backup plans including smaller tube size and adjuncts such as bougie, video laryngoscopy, and considers the possibility of needing anesthesia or other specialty personnel. These services are paged. Rapid Sequence Intubation is performed using rocuronium and a hemodynamic-preserving sedative such as etomidate. Intubation is challenging due to airway swelling, but is successful with smaller endotracheal tube size and video laryngoscopy or use of a bougie. After airway stabilization and adequate ventilation is established with either bag valve or ventilator support, the team identifies that the patient is hemodynamically stable by blood pressure, but initiates fluid resuscitation for burn management.

The secondary survey identifies a distended, rigid abdomen. A FAST exam is performed which reveals free fluid in the pelvis. Trauma surgery is paged for surgical management. Total body surface area of burn is calculated, and a circumferential burn of the upper extremity is identified. When lab work returns, the team leader interprets the elevated lactate and severe gap acidemia to suggest that the patient is likely suffering from cyanide poisoning. The team leader orders a CyanoKit to be administered empirically. The team leader also identifies that the patient has Carbon Monoxide poisoning, and should be kept on 100% FiO_2,_ but acknowledges that the patient is too unstable for transfer to hyperbarics at this time. Once treatment for cyanide is initiated, the surgical team arrives for transport to the operating room. The team leader gives a comprehensive sign out.

**Anticipated Mistakes:**

- **Failure to anticipate a difficult airway:** We found that teams typically recognized the need to intubate early, but did not routinely verbalize that a difficult airway was anticipated given the possibility of intraoral burns and mucosal swelling. We included the sizing of pediatric intubation equipment and pediatric variations of airway adjuncts as an important highlight in debriefing.
- **Use of Succinylcholine**: In general, rocuronium should be the preferred intubation agent in children. Especially in the setting of burns, use of succinylcholine should be avoided due to risk of hyperkalemia, which can cause rapid destabilization of hemodynamic status, cardiac arrhythmia, and arrest^1^. In the event of succinylcholine use, the patient proceeded to unstable ventricular tachycardia and cardiac arrest.
- **Premature closure**: The team may become preoccupied with transferring the patient for operative management and miss metabolic derangements of CN or CO toxicity. In these instances, the nurse confederate would prompt the team by saying that the trauma team was not yet available, and asked if there were additional treatments that could be pursued to stabilize the patient in the meantime.
- **Failure to recognize hemoperitoneum:** The team may be distracted by other injuries including burns, and fail to recognize the sequelae of blunt trauma. The team will likely ask for surgical consultation regardless. This can be mitigated by the facilitator stating that the surgical team is unavailable at this time, but requests a complete primary and secondary survey be completed with medical optimization prior to their arrival. Additionally, the facilitator should announce that the abdomen is tense and distended when it is examined during the surveys.
- **Uncertainty about treatment for cyanide toxicity:** The identification of cyanide toxicity is difficult as it is made empirically solely by metabolic derangements and clinical suspicion due to circumstances. If the team does not identify CN toxicity, this should be highlighted in the debrief to ensure that participants recognize the need to keep a high level of clinical suspicion in the setting of entrapped fire victims where synthetic materials are burning. If the team identifies a concern for CN toxicity but is uncertain of treatment, the nurse can offer to call poison control, and the facilitator can offer suggestions as this consultant.
- **Uncertainty about treatment for carbon monoxide poisoning:** The identification of carbon monoxide toxicity requires clinical suspicion on the team’s part due to circumstances. If the team does not identify CO toxicity, this should be highlighted in the debrief to ensure that participants recognize the need to keep a high level of clinical suspicion in the setting of entrapped fire victims, especially with prolonged extrication. If the team identifies a concern for CO toxicity but is uncertain of management or how to triage treatment, especially the possible need for hyperbaric therapy in the setting of other surgical concerns, the nurse can offer to call poison control, and the facilitator can offer suggestions as this consultant.

1. Martyn JAJ, Richtsfeld M, Warner DO. Succinylcholine-induced Hyperkalemia in Acquired Pathologic States: Etiologic Factors and Molecular Mechanisms. *Anesthesiology*. 2006;104(1):158-169. doi:[10.1097/00000542-200601000-00022](https://doi.org/10.1097/00000542-200601000-00022)
