# Supplementary material for: Pediatric Polytrauma Fire Victim Simulation
Source: MedEdPORTAL. 2024 Feb 27;20:11383. doi: 10.15766/mep_2374-8265.11383 (PMC10897059; doi:10.15766/mep_2374-8265.11383)
Supplement: Supplementary file 1 — Polytrauma Fire Sim Case.docxSim Environment Checklist.docxEKG, CXR, FAST, and Labs.docxPolytrauma Fire Debriefing Guide.docxPolytrauma Fire Victim Sim Survey.docxPolytrauma Debriefing.pptxPolytrauma Reference Sheet.docx [file mep_2374-8265.11383-s001.zip › B. Sim Environment Checklist.docx]

**Appendix B:** Simulation Scenario Environment Checklist

Instructions: This appendix should be used in advance of simulation during setup and preparation to ensure presence of all necessary equipment and to ensure an appropriately immersive environment.

| **Manikins** | Laerdal SimJunior ® or similar manikin representative of young school-aged child. The airway should be on the difficult intubation setting. Pulmonary settings should include inspiratory stridor and wheeze. |
| --- | --- |
| **Moulage/appearance** | 2^nd^ or 3^rd^ degree burns to 20% of body including face, chest, arms. There is a circumferential burn to the right forearm. There is soot around the mouth and nares. Lips are bright red. There is a seatbelt sign on the abdomen. |
| **Procedural Equipment** | - Cardiorespiratory monitor - Pulse oximetry - End tidal CO_2_ monitor and tubing - Supplemental oxygen (pediatric bag valve mask, nonrebreather) - First line intubation equipment (direct laryngoscopy blade and handle, endotracheal tube of appropriate size and smaller size, stylet, colorimeter, syringe) - Second line intubation equipment (video laryngoscope, pediatric bougie) - Defibrillator |
| **Medications** | Rapid Sequence Intubation Medications   - Etomidate - Rocuronium - Succinylcholine - Ketamine - Fentanyl - Versed   Cyanide Toxicity Treatment   - Cyanokit (hydroxocobalamin) – bright red fluid vial   Hyperkalemia Management   - Normal Saline bolus - Calcium Gluconate - Sodium Bicarbonate |
