# Supplementary material for: Pediatric Polytrauma Fire Victim Simulation
Source: MedEdPORTAL. 2024 Feb 27;20:11383. doi: 10.15766/mep_2374-8265.11383 (PMC10897059; doi:10.15766/mep_2374-8265.11383)
Supplement: Supplementary file 1 — Polytrauma Fire Sim Case.docxSim Environment Checklist.docxEKG, CXR, FAST, and Labs.docxPolytrauma Fire Debriefing Guide.docxPolytrauma Fire Victim Sim Survey.docxPolytrauma Debriefing.pptxPolytrauma Reference Sheet.docx [file mep_2374-8265.11383-s001.zip › F. Polytrauma Debriefing.pptx]

## Slide 1
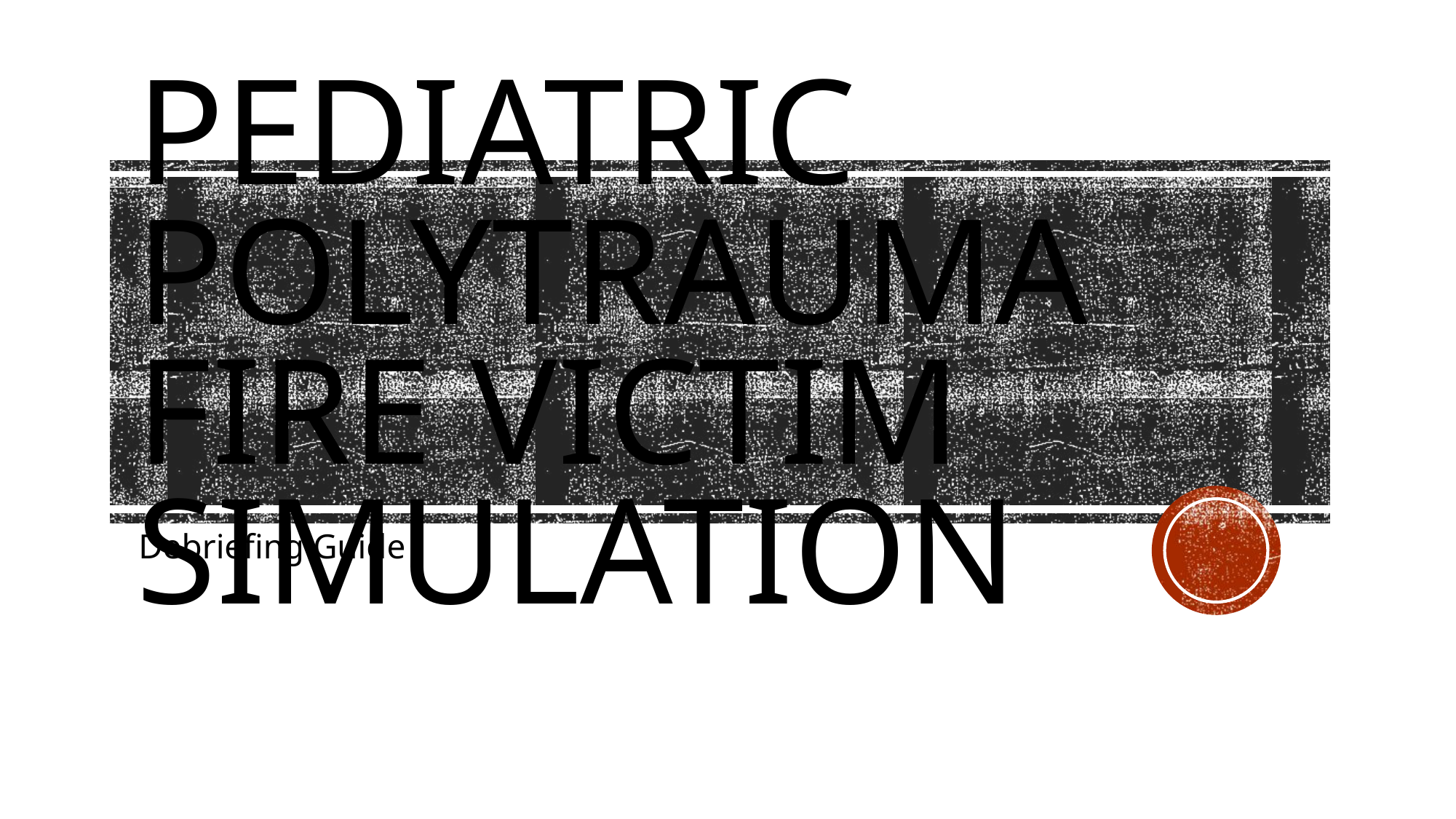

# Pediatric Polytrauma Fire Victim Simulation
Debriefing Guide

## Slide 2
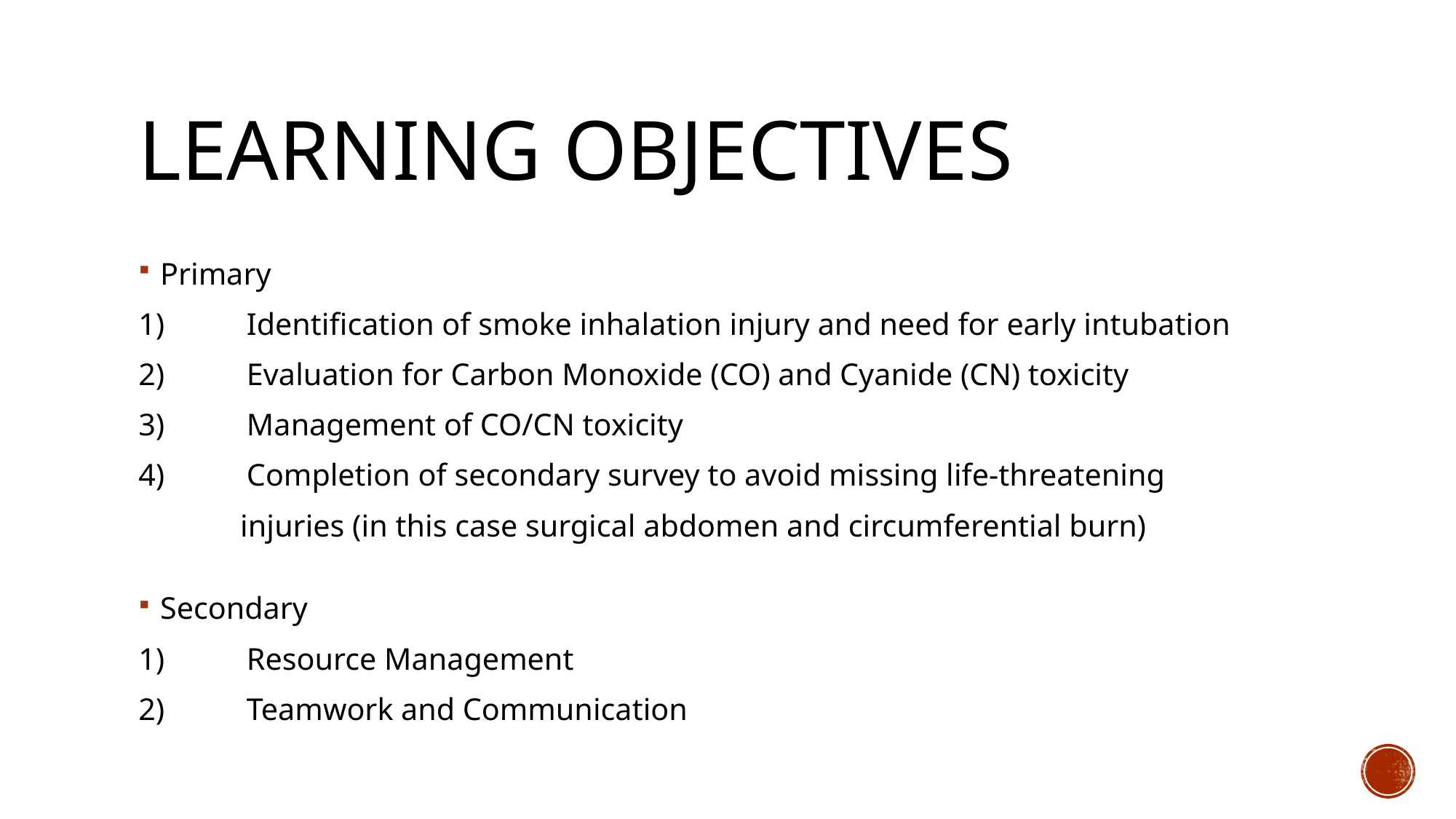

# Learning Objectives
Primary
1)	Identification of smoke inhalation injury and need for early intubation
2)	Evaluation for Carbon Monoxide (CO) and Cyanide (CN) toxicity
3)	Management of CO/CN toxicity
4)	Completion of secondary survey to avoid missing life-threatening
 injuries (in this case surgical abdomen and circumferential burn)
Secondary
1)	Resource Management
2)	Teamwork and Communication

## Slide 3
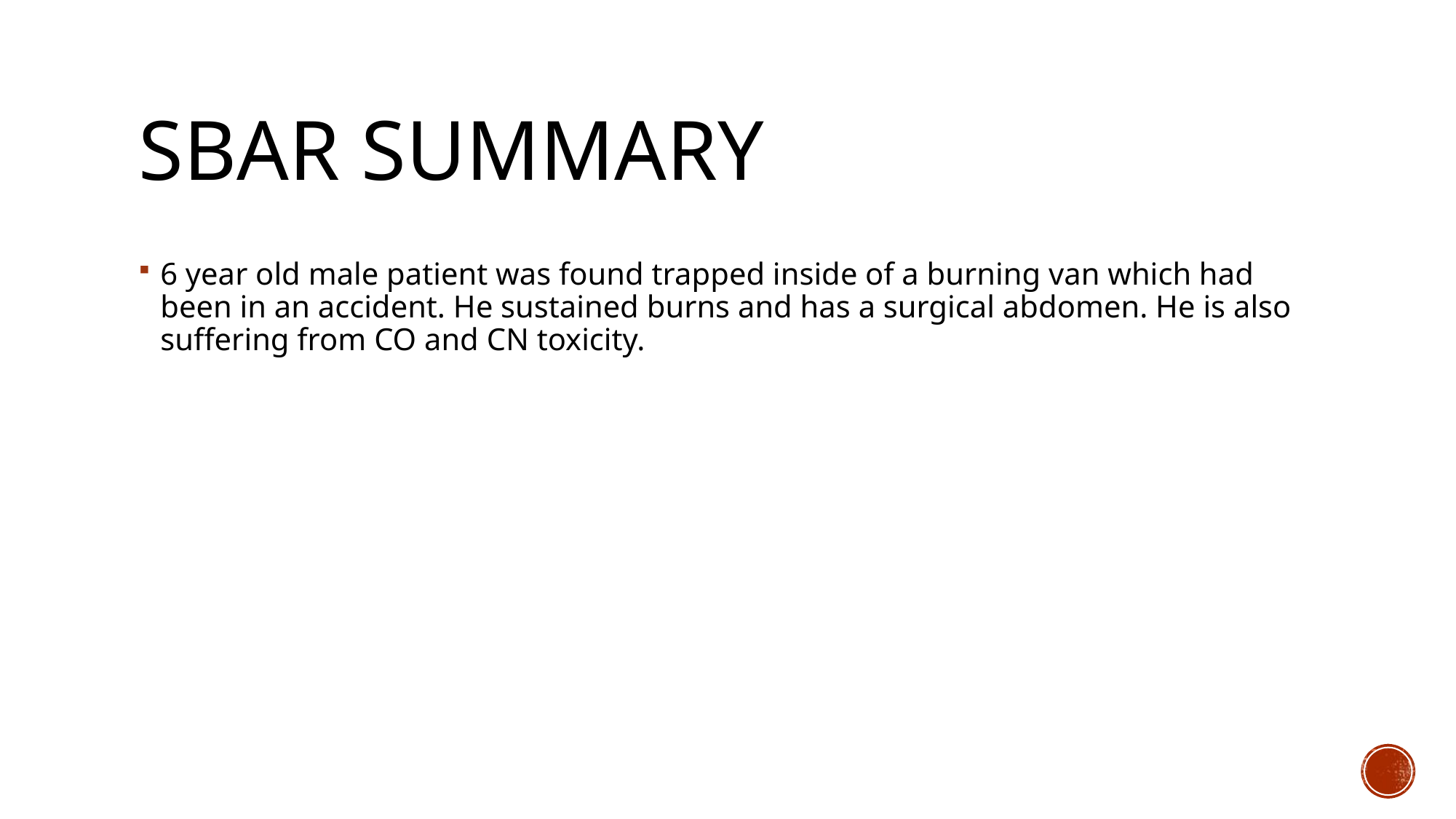

# SBAR Summary
6 year old male patient was found trapped inside of a burning van which had been in an accident. He sustained burns and has a surgical abdomen. He is also suffering from CO and CN toxicity.

## Slide 4
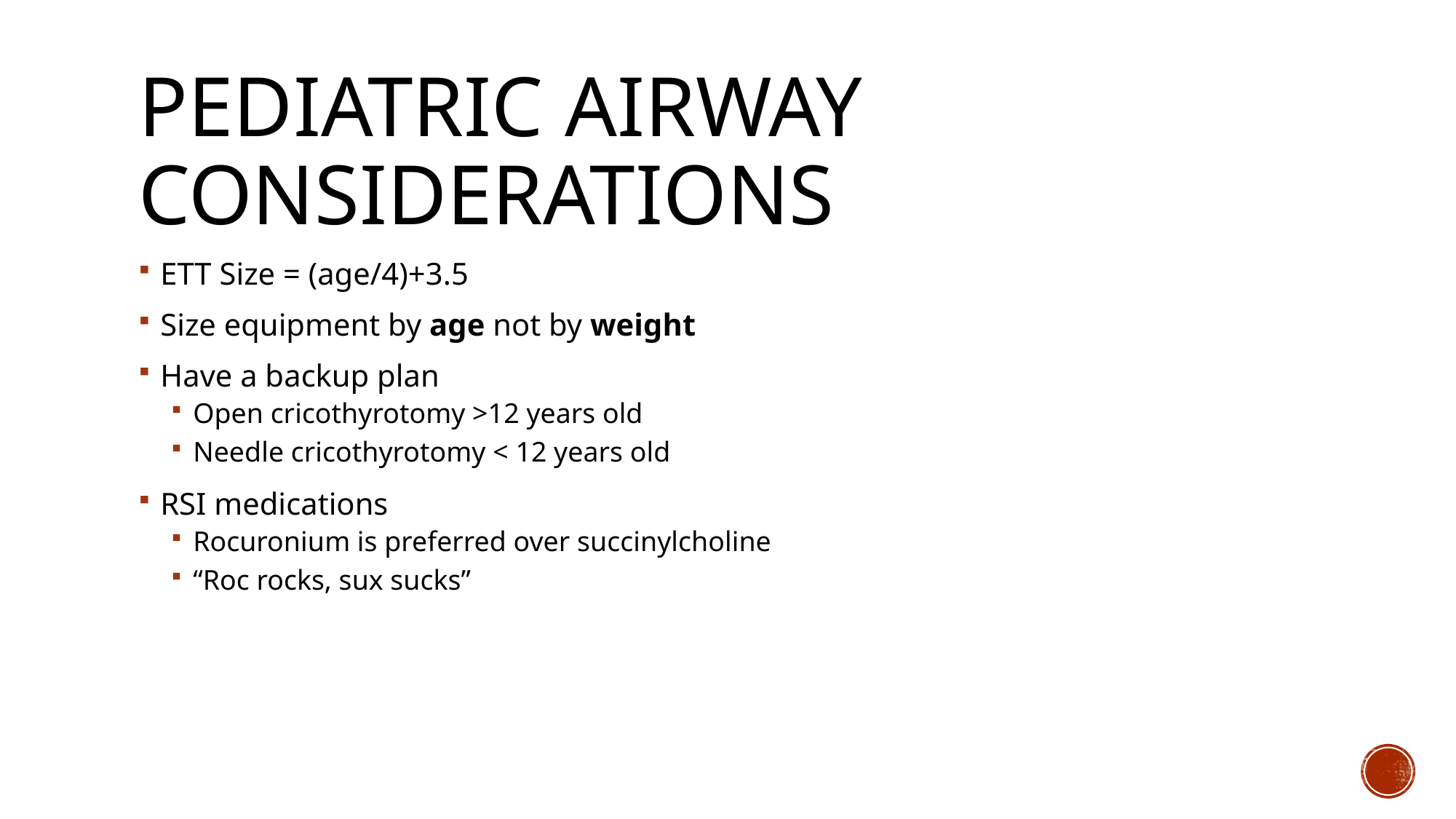

# Pediatric Airway Considerations
ETT Size = (age/4)+3.5
Size equipment by age not by weight
Have a backup plan
Open cricothyrotomy >12 years old
Needle cricothyrotomy < 12 years old
RSI medications
Rocuronium is preferred over succinylcholine
“Roc rocks, sux sucks”

## Slide 5
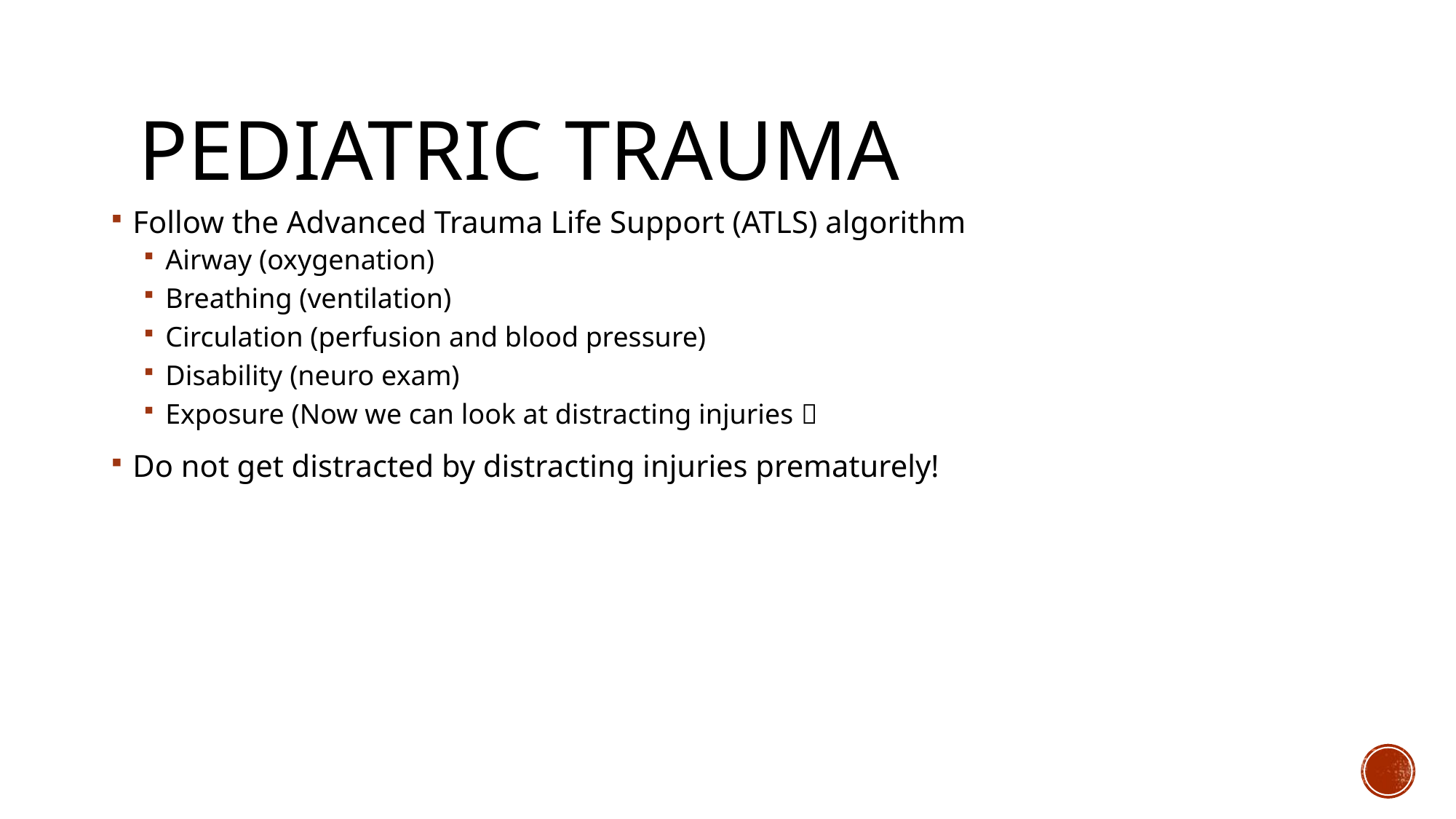

# Pediatric Trauma
Follow the Advanced Trauma Life Support (ATLS) algorithm
Airway (oxygenation)
Breathing (ventilation)
Circulation (perfusion and blood pressure)
Disability (neuro exam)
Exposure (Now we can look at distracting injuries 
Do not get distracted by distracting injuries prematurely!

## Slide 6
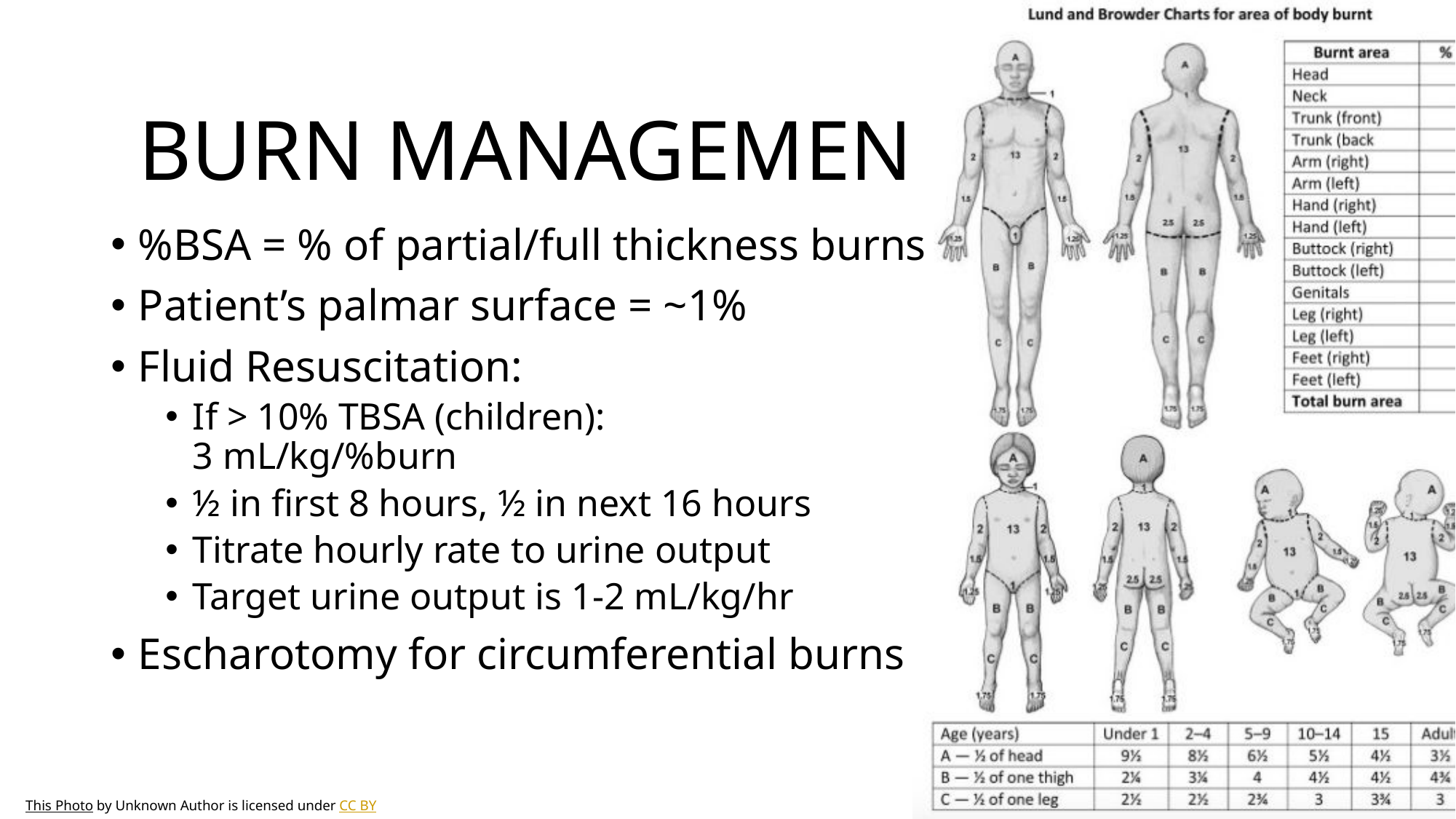

# Burn Management
%BSA = % of partial/full thickness burns
Patient’s palmar surface = ~1%
Fluid Resuscitation:
If > 10% TBSA (children):
3 mL/kg/%burn
½ in first 8 hours, ½ in next 16 hours
Titrate hourly rate to urine output
Target urine output is 1-2 mL/kg/hr
Escharotomy for circumferential burns
This Photo by Unknown Author is licensed under CC BY

## Slide 7
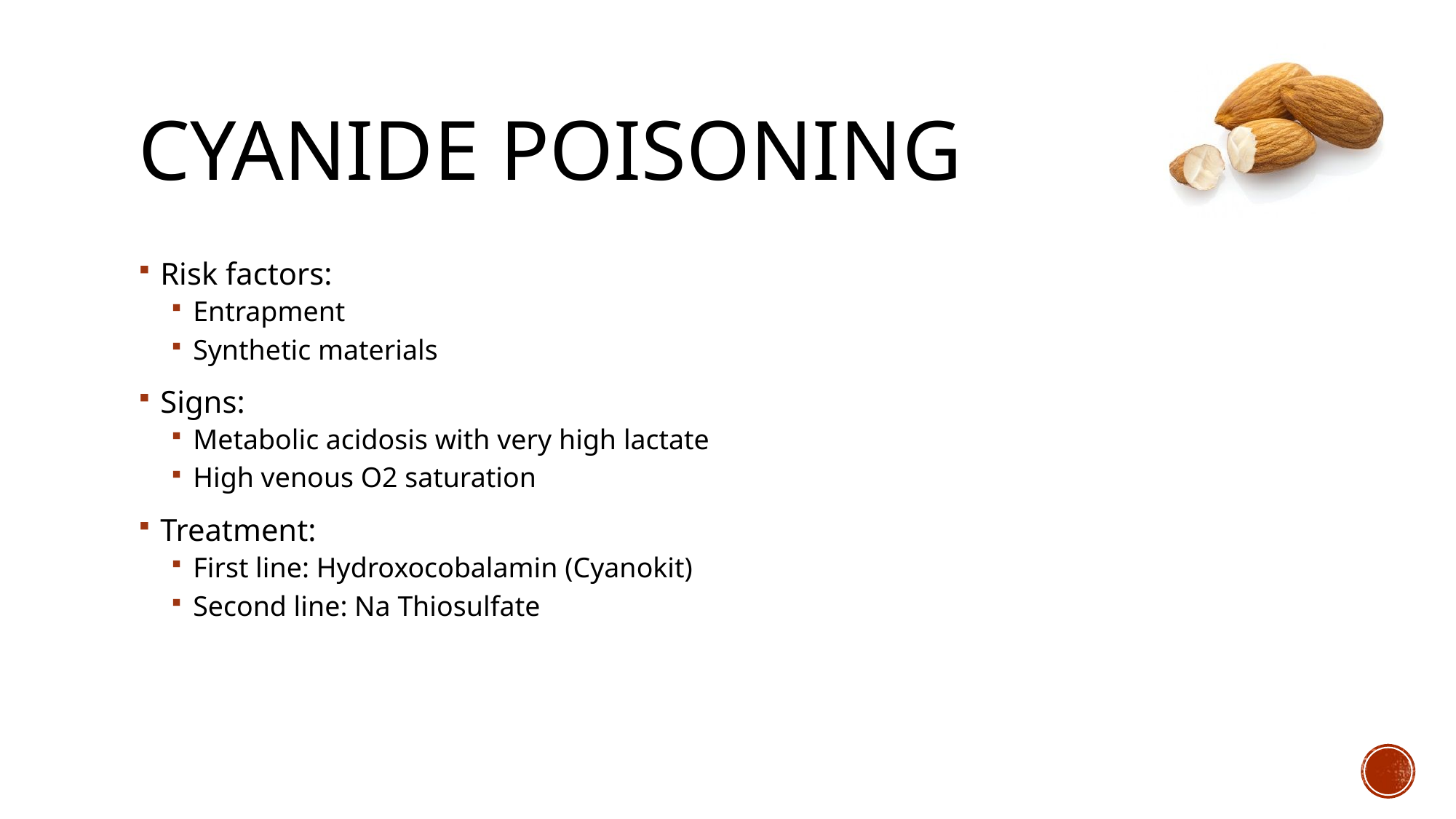

# Cyanide Poisoning
Risk factors:
Entrapment
Synthetic materials
Signs:
Metabolic acidosis with very high lactate
High venous O2 saturation
Treatment:
First line: Hydroxocobalamin (Cyanokit)
Second line: Na Thiosulfate

## Slide 8
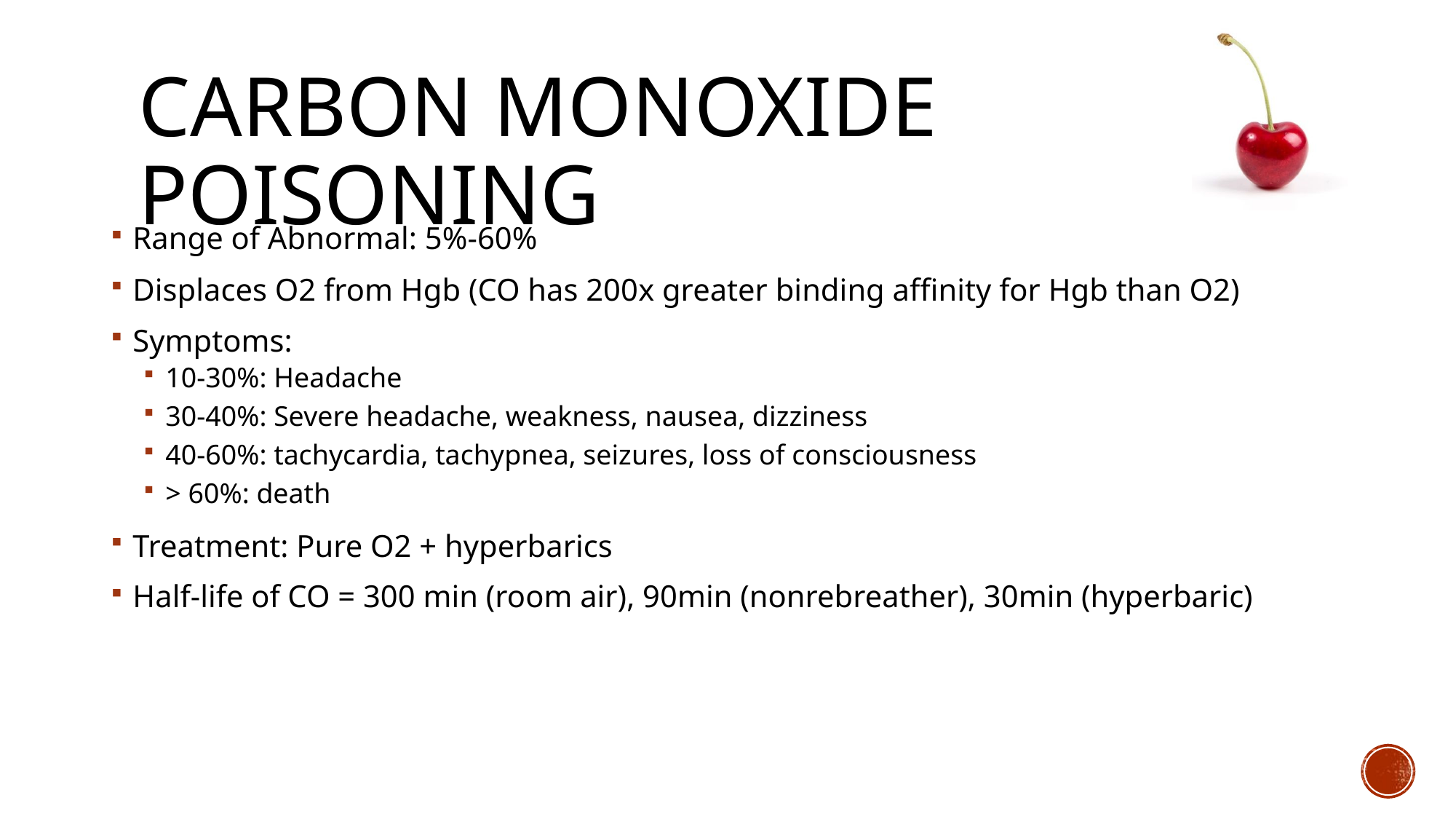

# Carbon Monoxide Poisoning
Range of Abnormal: 5%-60%
Displaces O2 from Hgb (CO has 200x greater binding affinity for Hgb than O2)
Symptoms:
10-30%: Headache
30-40%: Severe headache, weakness, nausea, dizziness
40-60%: tachycardia, tachypnea, seizures, loss of consciousness
> 60%: death
Treatment: Pure O2 + hyperbarics
Half-life of CO = 300 min (room air), 90min (nonrebreather), 30min (hyperbaric)

## Slide 9
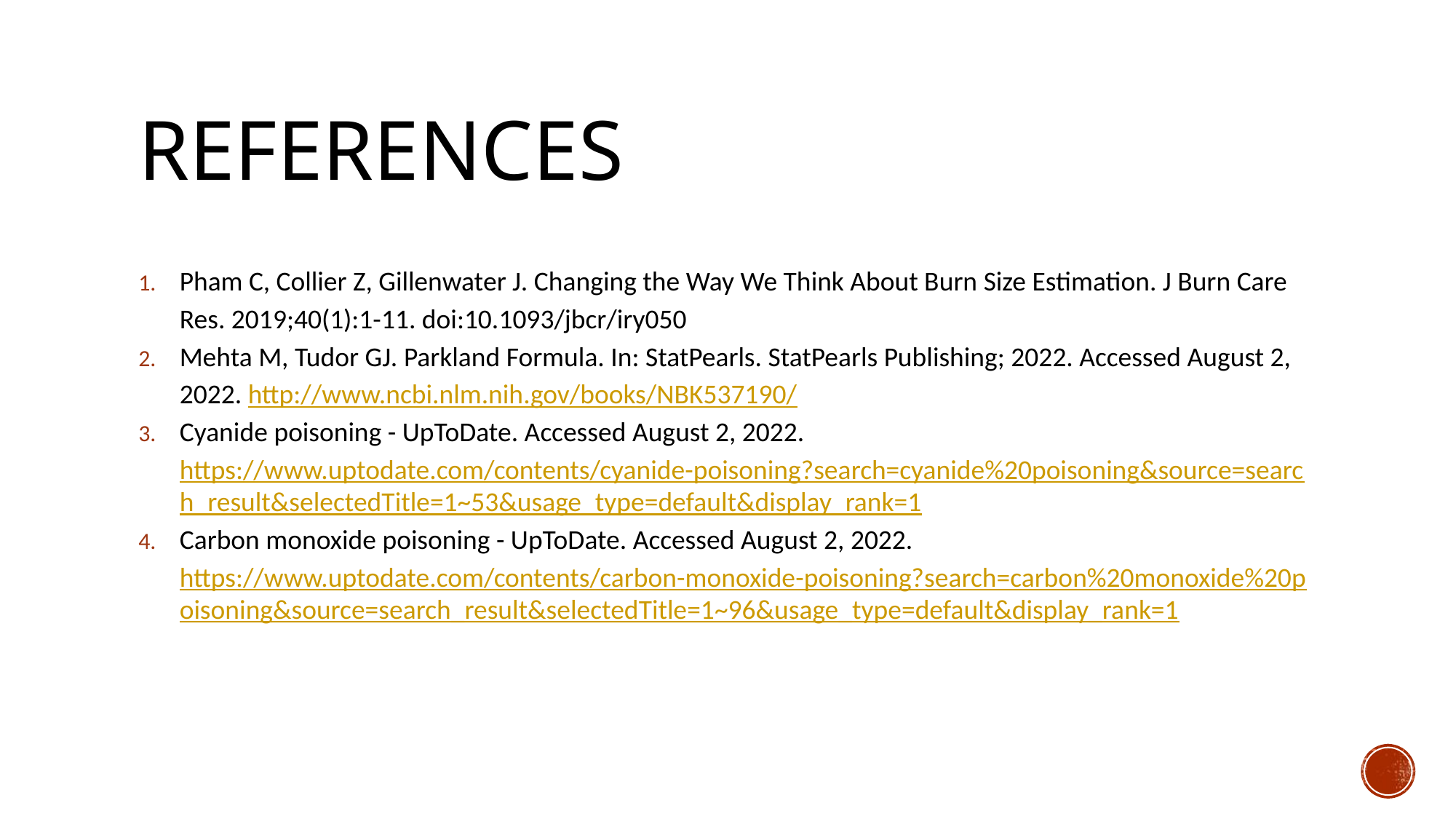

# References
Pham C, Collier Z, Gillenwater J. Changing the Way We Think About Burn Size Estimation. J Burn Care Res. 2019;40(1):1-11. doi:10.1093/jbcr/iry050
Mehta M, Tudor GJ. Parkland Formula. In: StatPearls. StatPearls Publishing; 2022. Accessed August 2, 2022. http://www.ncbi.nlm.nih.gov/books/NBK537190/
Cyanide poisoning - UpToDate. Accessed August 2, 2022. https://www.uptodate.com/contents/cyanide-poisoning?search=cyanide%20poisoning&source=search_result&selectedTitle=1~53&usage_type=default&display_rank=1
Carbon monoxide poisoning - UpToDate. Accessed August 2, 2022. https://www.uptodate.com/contents/carbon-monoxide-poisoning?search=carbon%20monoxide%20poisoning&source=search_result&selectedTitle=1~96&usage_type=default&display_rank=1
