# Supplementary material for: Pediatric Polytrauma Fire Victim Simulation
Source: MedEdPORTAL. 2024 Feb 27;20:11383. doi: 10.15766/mep_2374-8265.11383 (PMC10897059; doi:10.15766/mep_2374-8265.11383)
Supplement: Supplementary file 1 — Polytrauma Fire Sim Case.docxSim Environment Checklist.docxEKG, CXR, FAST, and Labs.docxPolytrauma Fire Debriefing Guide.docxPolytrauma Fire Victim Sim Survey.docxPolytrauma Debriefing.pptxPolytrauma Reference Sheet.docx [file mep_2374-8265.11383-s001.zip › G. Polytrauma Reference Sheet.docx]

**Appendix G**: Participant Reference Sheet

Instructions: This is meant to be handed out to participants either before or after the debriefing as a take-home physical reminder of key learning points from the case.

| **Pediatric Polytrauma Fire Victim – Participant Reference Sheet** |
| --- |
| This was a 6-year-old boy brought in by EMS after extraction from a school van accident. He was entrapped in the vehicle and exposed to smoke and fire. His injuries included an unstable airway, partial thickness circumferential burns, hemoperitoneum, and metabolic derangements consistent with carbon monoxide and cyanide toxicity. |
| **Pediatric Airway Considerations** |
| Always size equipment by age, not by weight  Endotracheal Tube (ETT) Sizing   - Age/4 + 3.5 (cuffed – always use cuffed if available) - Be prepared with smaller size if risk of airway edema - 1 x ETT = (age/4) + 3.5 [Cuffed] - 2 x ETT = Naso- or orogastric tube or foley size - 3 x ETT = ETT depth - 4 x ETT = maximum chest tube size   Rapid Sequence Intubation   - Rocuronium is preferred over succinylcholine in pediatric patients   Backup airway plans   - Call for help early! - Video Laryngoscopy, bougie, fiberoptic - Open cricothyrotomy if above 12 years old - Needle cricothyrotomy if younger than 12 years old - Reverse intubation |
| **Pediatric Trauma and Burns** |
| - %BSA = body surface area percentage of partial/full thickness burns only - Palmar surface area = 0.5-2% depending on BMI^1^ - Assess for circumferential burns which will require escharotomy - Fluid Resuscitation^2^:   - If > 10% TBSA (children): 3 mL/kg/%burn   - ½ in first 8 hours, ½ in next 16 hours   - Titrate hourly rate to urine output   - Target urine output is 1-2 mL/kg/hr   - Include maintenance fluids - Normal vitals in children   - Reference PALS cards or phone apps   - Minimum systolic BP = 70 + (age*2) - CPR in children   - Rate: 100-120 BPM   - 15:2 with 2 people   - Continuous with advanced airway |
| **Management of Cyanide Poisoning^3^** |
| - Empiric CN poisoning treatment in a metabolic acidosis with high lactate   - Caused by victim entrapment in a fire where synthetic materials are combusting   - Lab findings: anion gap metabolic acidosis, high lactate, high venous O2 sat   - Caused by: Electron transport chain uncoupling 🡪 metabolic (lactic) acidosis   - Treatment:     - Hydroxocobalamin (Cyanokit) – binds CN to form B12. Ideal, but bright red and will affect lab work, so draw off blood for labs before starting treatment     - Secondary treatment: Na Thiosulfate.     - Avoid Amyl and Na Nitrite- Converts Hgb to methemoglobin; CN preferentially binds methemoglobin. But this causes methemoglobinemia & hypotension.     - Do not delay treatment by waiting for lab results. Treat empirically if clinical evidence of CN poisoning. |
| **Management of Carbon Monoxide Poisoning^4^** |
| - Range of Abnormal: 5%-60% - Caused by: Displaces O_2_ from Hgb (CO has 200x greater binding affinity for Hgb than O_2_) - Can cause false elevation in SpO2 level due to the inability of SpO2 monitors to differentiate between oxyhemoglobin and carboxyhemoglobin - Symptoms:   - 10-30%: Headache   - 30-40%: Severe headache, weakness, nausea, dizziness   - 40-60%: tachycardia, tachypnea, seizures, loss of consciousness   - > 60%: death - Treatment: Pure O_2_ + hyperbarics - Half-life of CO = 300 min (room air), 90min (nonrebreather), 30min (hyperbaric) - Hyperbaric if persistent metabolic acidosis, altered mental status, myocardial infarction, CO >25% (10% if pregnant) |

1. Pham C, Collier Z, Gillenwater J. Changing the Way We Think About Burn Size Estimation. J Burn Care Res. 2019;40(1):1-11. doi:10.1093/jbcr/iry050
2. Mehta M, Tudor GJ. Parkland Formula. In: StatPearls. StatPearls Publishing; 2022. Accessed August 2, 2022. <http://www.ncbi.nlm.nih.gov/books/NBK537190/>
3. Cyanide poisoning - UpToDate. Accessed August 2, 2022. <https://www.uptodate.com/contents/cyanide-poisoning?search=cyanide%20poisoning&source=search_result&selectedTitle=1~53&usage_type=default&display_rank=1>
4. Carbon monoxide poisoning - UpToDate. Accessed August 2, 2022. <https://www.uptodate.com/contents/carbon-monoxide-poisoning?search=carbon%20monoxide%20poisoning&source=search_result&selectedTitle=1~96&usage_type=default&display_rank=1>
