# Supplementary material for: Pediatric Polytrauma Fire Victim Simulation
Source: MedEdPORTAL. 2024 Feb 27;20:11383. doi: 10.15766/mep_2374-8265.11383 (PMC10897059; doi:10.15766/mep_2374-8265.11383)
Supplement: Supplementary file 1 — Polytrauma Fire Sim Case.docxSim Environment Checklist.docxEKG, CXR, FAST, and Labs.docxPolytrauma Fire Debriefing Guide.docxPolytrauma Fire Victim Sim Survey.docxPolytrauma Debriefing.pptxPolytrauma Reference Sheet.docx [file mep_2374-8265.11383-s001.zip › _Educational Summary Report_11383.pdf]

MedEdPORTAL® The AAMC Journal of Teaching and Learning Resources

Original Publication Open Access

Pediatric Polytrauma Fire Victim Simulation

Lauren Vrablik, MD*, Robyn Wing, MD, MPH

*Corresponding author: l.s.vrablik@gmail.com

Abstract

Introduction: Pediatric trauma has long been one of the primary contributors to pediatric mortality. There are multiple cases in the literature involving cyanide (CN) toxicity, carbon monoxide (CO) toxicity, and smoke inhalation with thermal injury, but none in combination with mechanical trauma. **Methods:** In this 45-minute simulation case, emergency medicine residents and fellows were asked to manage a pediatric patient with multiple life-threatening traumatic and metabolic concerns after being extracted from a van accident with a resulting fire. Providers were expected to identify and manage the patient's airway, burns, hemoperitoneum, and CO and CN toxicities. **Results:** Forty learners participated in this simulation, the majority of whom had little prior clinical experience managing the concepts highlighted in it. All agreed or strongly agreed that the case was relevant to their work. After participation, learner confidence in the ability to manage each of the learning objectives was high. One hundred percent of learners felt confident or very confident in managing CO toxicity and completing primary and secondary surveys, while 97% were similarly confident in identifying smoke inhalation injury, preparing for a difficult airway, and managing CN toxicity. **Discussion:** This case was a well-received teaching tool for the management of pediatric trauma and metabolic derangements related to fire injuries. While this specific case represents a rare clinical experience, it is within the scope of expected knowledge for emergency medicine providers and offers the opportunity to practice managing multisystem trauma.

Keywords
Carbon Monoxide, Cyanide, Polytrauma, Thermal Injury, Trauma Triage, Emergency Medicine, Medical Toxicology, Pediatric Emergency Medicine, Simulation

Educational Objectives

By the end of this activity, learners will be able to:

1. Identify critical airway caused by smoke inhalation injury.
2. Prepare for a difficult pediatric airway using video techniques.
3. Manage carbon monoxide toxicity using oxygen.
4. Identify cyanide toxicity through situational exposure and metabolic derangements.
5. Manage cyanide toxicity using the Cyanokit.
6. Complete primary and secondary survey assessments to avoid missing injuries, including hemoperitoneum and circumferential burns.

Citation:
Vrablik L, Wing R. Pediatric polytrauma fire victim simulation. *MedEdPORTAL*. 2024;20:11383.
https://doi.org/10.15766/mep_2374-8265.11383

Introduction

Pediatric trauma has long been one of the primary contributors to pediatric mortality. The Centers for Disease Control and Prevention reported in 2018 that unintentional injury was the number one cause of mortality in all pediatric patients greater than 1 year of age.[1] Unintentional injury comprises many subcategories, with motor vehicle injury and fire or burn injury ranking among the top three causes of death for children under 14 years of age.[1] Although these injuries are a rare presentation to the emergency department (polytrauma and burns represented 0.7% and 0.4%, respectively, of all emergency department visits in 2017),[2] they represent a significant majority of deaths in children, and their management warrants careful understanding of best practices.

Education can be delivered in many forms, with clinical teaching being the primary means of educating physician trainees. However, clinical exposure to pediatric trauma is more limited than adult trauma due to the relative paucity of pediatric trauma cases compared to adult counterparts.[3] Indeed, learning management of pediatric trauma has been reported by trainees as being one of their greatest perceived deficits.[4] Therefore,

Copyright © 2024 Vrablik and Wing. This is an open-access publication distributed under the terms of the Creative Commons Attribution license.

alternative methods must be engaged to ensure competency of emergency medicine (EM) and pediatric providers in this domain in independent clinical practice.

Simulation is well established as an effective and indispensable teaching modality in medical education.[5-9] Medical simulation for the training of trauma management has proven to be an effective method of gaining and maintaining clinical competency.[10] In a review of current *MedEdPORTAL* publications, we found cases involving cyanide (CN) toxicity, carbon monoxide (CO) toxicity, and smoke inhalation with thermal injury,[11,12] but none in combination with mechanical trauma.[13,14]

Here, we present a simulation case of a pediatric patient with multiple life-threatening traumatic and metabolic concerns after being extracted from a car accident with a resulting fire. Providers are expected to manage the patient's airway compromise, burns, hemoperitoneum, and CO and CN toxicities. This simulation aims to challenge senior EM trainees with a complex case that assesses their ability to comanage multiple life-threatening medical problems in a single trauma victim.

Methods

Development

We developed this case (Appendix A) as part of a mandatory simulation curriculum for EM and pediatric EM (PEM) training programs. Participants were recruited during didactic sessions. The target population was emergency department providers, including attending, fellow, and resident physicians and advanced practice practitioners. However, given in-person attendance restrictions during COVID protocols, only EM and PEM physicians participated. The case was preliminarily disclosed to be an advanced management case, and the facilitators encouraged senior trainees to act as team leaders. In order to maximize the learning potential of participating in the case, participants had to have prerequisite comfort in identifying abnormal vital signs, interpreting point-of-care ultrasound, and managing abnormal labs. For the purposes of this case, we considered this to be anyone beyond year 1 of training.

Equipment/Environment

The setting for this simulation case was a medical simulation center, which emulated a standard resuscitation bay in an emergency department. The equipment and medications available were listed in the simulation scenario environment checklist (Appendix B). The case was conducted using a high-fidelity Laerdal SimJunior manikin, and vital signs were demonstrated on a monitor using standard LLEAP (Laerdal Learning Application) simulation software. Moulage was critical to case fidelity and included 20% body surface area burns, including a circumferential burn of the right forearm. Additionally, the manikin had soot around the mouth and nares, red lips, and a seatbelt sign on the abdomen. Bruising and burns were created using oil-based simulation manikin moulage makeup placed over Tegaderm for texture and to preserve the manikin. Soot around the mouth and nose was actual soot taken from a fire. Laboratory results and diagnostic modalities, including chest X-ray, EKG, and a bedside Focused Assessment with Sonography for Trauma (FAST) image, were available to learners upon request (Appendix C).

Personnel

The personnel needed to implement this case included a faculty instructor, a simulation technician, and embedded participants or facilitators playing the roles of an emergency medical technician (EMT), nurse, and respiratory therapist (RT; Appendix A). Ideally, there were four case operators—technician, facilitator, nurse, and one person playing the role of emergency medical services (EMS) and RT. At a minimum, there had to be a facilitator, nurse, and simulation technician. Embedded participants were PEM physician faculty or fellows involved in the development of this case. A simulation technician operated the manikin, including vital sign changes and manikin physiology. The technician monitored for successful bag valve mask ventilation and subsequent intubation. The RT assisted in intubation setup and postintubation care but did not perform the intubation or make any suggestions for equipment. Three to five learners could participate in the case.

Implementation

We implemented this simulation during scheduled EM didactics in the simulation center, with four learners on each team. All active simulation participants were present in person. The simulated case ran for approximately 15 minutes, and we devoted an additional 30 minutes to debriefing. The team ran the simulation as though they had the resources of a level 1 trauma center. The SimJunior manikin was ready on the gurney in full moulage, including seatbelt sign, burns, and soot to the face, without any IV access or monitoring equipment in place. A white sheet was placed over the manikin to indicate that the patient had not arrived yet. The team was called to the bedside via an overhead page saying, "EMS arrival to the critical care bay," at which time the sheet was removed to reveal the manikin.

The EMT and nurse were at the bedside when the team arrived. The EMT was appropriate and helpful but stressed. They gave the learner team a brief overview of the accident scene from which the child had been removed, stating that they were called

Copyright © 2024 Vrablik and Wing. This is an open-access publication distributed under the terms of the Creative Commons Attribution license.

to a school van accident involving a head-on collision. There were multiple casualties found on scene. Extrication took 20 minutes due to an active fire. The child was found trapped inside with significant smoke. No resuscitation was performed on scene, but a nonrebreather was applied immediately. En route, the patient was initially awake and coughing, then developed stridor and became obtunded just prior to arrival. IV access was not obtained given extremity burns, and no medications were given. The nurse was a seasoned veteran of trauma care and performed all tasks requested but did not offer any suggestions.

The learner team ran the resuscitation, and the manikin responded according to the progression in Appendix A. On primary survey, learners were expected to identify the immediate need for intubation and anticipate a difficult pediatric airway. A postintubation chest X-ray (Appendix C) was provided showing adequate placement if the manikin was successfully intubated. On secondary survey, learners were expected to identify hemoperitoneum as elicited by a seatbelt sign on the abdomen with corresponding positive FAST ultrasound (Appendix C). If requested, the surgical trauma team was not available for bedside evaluation until 15 minutes into the case. Learners were expected to identify CO and CN poisoning by requesting appropriate lab studies given clinical suspicion due to altered mental status in an entrapped fire victim. Lab values (Appendix C) reflected a severe lactic acidosis concerning for CN toxicity, as well as an elevated CO level. The nurse also remarked that the blood appeared bright red when the team requested a blood draw. Treatment options for CN poisoning included a Cyanokit, while treatment for CO poisoning was limited to 100% oxygen due to competing needs for surgical management. (The facilitator later discussed indications for hyperbaric treatment during the debriefing.) At 15 minutes, the surgical team became available to take sign-out on the patient. After the team leader summarized the key findings, interventions, and proposed plan, the case ended.

Immediately following the simulation, learners engaged in a 30-minute debrief (Appendix D) and completed a survey (Appendix E). The debrief was structured for time to cover all learning objectives addressed in the accompanying PowerPoint (Appendix F), which was available as a visual aid to supplement the debrief. In one iteration, there were learners observing remotely via video feed. These remote learners did not participate in the physical simulation but were encouraged to participate actively in the debrief. Critical learning objectives were also included in a participant reference sheet (Appendix G) for personal review at home.

Debriefing

This case was debriefed using a mix of scripted debriefing and the advocacy and inquiry approach[15] (Appendix D). The scripted content of the debrief followed the learning objectives in order of the expected progression of findings as the team should have discovered them by performing a primary and secondary survey. Scripting allowed for structured time management of this highly detailed debrief. Advocacy and inquiry were intertwined as the method of generating discussion to highlight correct and incorrect educational management strategies identified by facilitators during the simulation. Learners were asked to describe their intubation plan in detail, including pediatric considerations such as equipment sizing and endotracheal tube size cutoff for compatibility with a standard gum elastic bougie versus a pediatric bougie. Facilitators prompted learners to estimate the body surface area of burns for this patient and reviewed literature on fluid resuscitation principles. Debriefing prompts included a discussion of the risks and benefits of various CN poisoning antidotes, as well as a discussion of the physiology behind oxygen therapy versus hyperbaric treatment. Facilitators gave special consideration to the contraindications to hyperbarics in a patient with the potential for hemodynamic instability.

Assessment

The pediatric polytrauma fire victim case survey (Appendix E) was created to evaluate the level of confidence in the educational objectives after this teaching intervention, as perceived by the participants. Participants were advised of the study per standard consent protocol and were told that they would complete a survey (Appendix E) at the conclusion of debriefing. Appendix E was developed by adapting a previously published survey of a similarly complex case.[16] Our survey used standard Likert-scale questions regarding educational objectives being met and participant confidence. It also invited participants to explain how the case might influence their future practice and how it could be improved for future learners.

Results

Forty learners participated in this simulation and debriefing. Of this number, 33 participated in person, and seven viewed remotely. One hundred percent of participants submitted a response survey. Twenty-eight (70%) were residents in EM, six (15%) were attendings in PEM, and six (15%) were fellows in PEM. Previous self-reported experience with polytrauma fire victims, management of CN toxicity, management of CO toxicity, and inhalation injury was low, with the majority of participants citing caring for zero or one patient with these conditions previously. Prior experience for each learning

Copyright © 2024 Vrablik and Wing. This is an open-access publication distributed under the terms of the Creative Commons Attribution license.

objective was variable between learner groups, with attendings reporting the most experience, followed by EM residents, then PEM fellows.

In the debrief, many learners stated that they were challenged by the lack of immediate availability of surgical specialists. A significant amount of time was dedicated to the discussion of medical management of the metabolic derangements in this case. Facilitators found that learners sometimes became preoccupied by surgical complaints and failed to address concurrent metabolic derangements or identify and treat CN and CO poisonings.

There was very strong agreement with the case's realism and educational takeaways (Table 1), as well as strong confidence in the ability to perform the learning objectives after completing the simulation (Table 2). Unfortunately, after survey completion by one group, we determined that question 9, regarding the effectiveness of teaching the evaluation and management of smoke inhalation injuries and metabolic derangements (Appendix E), had been cut off of the paper copy. This was rectified for future groups. Therefore, the recorded number of responses for this question ($n = 28$) was lower than for the remaining ones (Table 1). The survey also asked for free-text comments and feedback. There were 31 responses to the question "What did you take away from this case and/or how will it change your practice?" We identified several themes. Sixteen responses (52%) indicated that the discussion of CN toxicity and management with Cyanokits was a primary takeaway. Nine responses (29%) cited assessment and management of an unstable airway. Some representative comments are listed below:

- "Loved the complexity and difficulty of the case. Would like more like this."
- "Always look for cyanide and other inhalation injuries."
- "Don't get side tracked by evident trauma on initial assessment, stick to ABCs."
- "Focus on primary survey and then move on even if there are distracting features such as burns."
- "Thinking about cyanide toxicity, securing the airway early, balancing inhalation injury with blunt trauma."

Participants also had the opportunity to provide constructive criticism but offered very little. Three participants suggested a longer or more detailed debrief.

Discussion

This case is an advanced scenario that challenges the learner to focus on triaging and managing a variety of life-threatening complications of blunt trauma and fire entrapment. Overall, the prior clinical experience of our learners in the learning objectives was low, highlighting a need for additional training in this content. Postsurvey scores showed exceptionally high levels of satisfaction with the content delivered and learner confidence despite the complexity and intensity of the case scenario. Learners did not seem intimidated by the level of difficulty, instead expressing appreciation for the opportunity to practice the skills in a safe environment. Specifically, learners were most challenged with the need to triage multiple competing medical and surgical needs. While trainees further along in their respective programs were selected as team leaders, they described a sense of challenge and productive stress from the case. Free-text feedback was positive and underscored sincere reflection on the intended learning objectives.

The debriefing session highlighted the importance of relying on standard trauma algorithms despite distracting injuries to optimize patient outcomes; however, it also encouraged participants to think beyond a trauma evaluation and consider medical resuscitation needs. From a trauma perspective, pediatric airway management was the most frequently referenced area of self-reflection. In the debrief, EM trainees felt the review of equipment sizing and rapid sequence intubation medication choices and how these could differ from the adult population was particularly helpful. For pediatric-trained EM fellows, the debrief focused more heavily on rarely encountered metabolic derangements of CN toxicity. Across groups, there was less emphasis on CO management and fluid resuscitation in burn

Table 1. Participant Agreement With Case Effectiveness

Statement[a]	*N*	*M* (*SD*)	% Agreeing or Strongly Agreeing
This simulation case provided is relative to my work.	40	5.0 (0.2)	100
This simulation case was realistic.	40	4.8 (0.4)	100
This simulation case was effective in teaching trauma resuscitation skills.	40	4.9 (0.3)	100
This simulation case was effective in teaching about evaluation and management of smoke inhalation injuries and metabolic derangements.	28	4.9 (0.3)	100
The debrief created a safe environment.	40	5.0 (0.2)	100
The debrief promoted reflection and team discussion.	40	5.0 (0.2)	100

[a]Rated on a 5-point Likert scale (1 = *strongly disagree*, 2 = *disagree*, 3 = *neither agree nor disagree*, 4 = *agree*, 5 = *strongly agree*).

Copyright © 2024 Vrablik and Wing. This is an open-access publication distributed under the terms of the Creative Commons Attribution license.

Table 2. Participant Confidence in Learning Objectives (*N* = 38)

Statement[a]	*M* (*SD*)	% Confident or Very Confident
After participating in this session, how confident are you in your ability to:		
Identify smoke inhalation injury and need for early intubation.	4.6 (0.6)	97
Recognize and prepare for a potential difficult airway.	4.6 (0.6)	97
Evaluate and manage carbon monoxide toxicity.	4.5 (0.5)	100
Evaluate and manage cyanide toxicity.	4.4 (0.5)	97
Complete primary and secondary survey to avoid missing life-threatening injuries.	4.7 (0.5)	100

[a]Rated on a 5-point Likert scale (1 = *very not confident*, 2 = *not confident*, 3 = *neutral*, 4 = *confident*, 5 = *very confident*).

management during the debrief, as guided by participant questions. However, all learning objectives generated active discussion.

There are inherent limitations to our case. Given COVID restrictions at our institution at the time, group sizes were limited. Although every effort was made to replicate the standard available resources, including personnel, in level 1 trauma, the inherent size limitation artificially reduced confounding factors, such as noise and limited space created by the larger trauma response teams more typically seen. This artificial reduction in background stimuli may have influenced participants' perceived confidence. While all simulation participants were in person, some observers completing the survey had experienced the simulation digitally via Zoom. Although both audio and video of the simulation and debrief were broadcast to this group, their experience was inherently different from that of the in-person participants. These differences were not probed in the debrief or survey and therefore cannot be commented on here. Another limitation is the outcome measure of learner confidence using Likert scales. While the Kirkpatrick level 1 and 2 data are preliminarily indicative of positive outcomes from this learning experience,[17] higher-level outcome data would be preferable, as confidence does not reflect true clinical ability. Lastly, inherent limitations exist with the use of simulation as a study methodology. These include human factors such as variability in embedded participant performance, electronic equipment failures, and limitations in moulage and in manikins' portrayal of physical exam findings.

Future directions for utilization and study of this simulation include conducting it in the emergency department setting to allow for multidisciplinary education. An in situ simulation could also assist in determining accessibility and availability of equipment and medications and allow for true testing of systems such as mobilizing consultation services like the trauma team.

In summary, we believe that this simulation is a high-acuity, low-frequency scenario that an emergency physician in any region could reasonably be asked to manage. Most children are cared for in community emergency settings without dedicated pediatric providers. Therefore, given the high degree of satisfaction seen with this case, we will continue to implement it as a regular part of both the PEM and EM training curricula at our institution.

Appendices

A. Polytrauma Fire Sim Case.docx
B. Sim Environment Checklist.docx
C. EKG, CXR, FAST, and Labs.docx
D. Polytrauma Fire Debriefing Guide.docx
E. Polytrauma Fire Victim Sim Survey.docx
F. Polytrauma Debriefing.pptx
G. Polytrauma Reference Sheet.docx

All appendices are peer reviewed as integral parts of the Original Publication.

Lauren Vrablik, MD: Third-Year Fellow, Division of Pediatric Emergency Medicine, Department of Emergency Medicine, Warren Alpert Medical School of Brown University; ORCID: https://orcid.org/0000-0002-6181-8266

Robyn Wing, MD, MPH: Associate Professor, Division of Pediatric Emergency Medicine, Departments of Emergency Medicine and Pediatrics, Warren Alpert Medical School of Brown University and Rhode Island Hospital/Hasbro Children's Hospital; Director of Pediatric Simulation, Lifespan Medical Simulation Center

Disclosures
None to report.

Funding/Support
None to report.

Ethical Approval
Reported as not applicable.

Copyright © 2024 Vrablik and Wing. This is an open-access publication distributed under the terms of the Creative Commons Attribution license.

References

1. WISQARS injury data. Centers for Disease Control and Prevention. Accessed January 4, 2024. https://www.cdc.gov/injury/wisqars/index.html
2. Rui P, Kang K. *National Hospital Ambulatory Medical Care Survey: 2017 Emergency Department Summary Tables*. National Center for Health Statistics; 2017. Accessed January 4, 2024. https://www.cdc.gov/nchs/data/nhamcs/web_tables/2017_ed_web_tables-508.pdf
3. Chang MC. *National Trauma Data Bank 2016: Annual Report*. American College of Surgeons; 2016. Accessed January 4, 2024. https://www.facs.org/media/ez1hpdcu/ntdb-annual-report-2016.pdf
4. Trainor JL, Krug SE. The training of pediatric residents in the care of acutely ill and injured children. *Arch Pediatr Adolesc Med*. 2000;154(11):1154-1159. https://doi.org/10.1001/archpedi.154.11.1154
5. Kester-Greene N, Hall AK, Walsh CM. Simulation curricular content in postgraduate emergency medicine: a multicentre Delphi study. *CJEM*. 2019;21(5):667-675. https://doi.org/10.1017/cem.2019.348
6. Hart D, Bond W, Siegelman JN, et al. Simulation for assessment of milestones in emergency medicine residents. *Acad Emerg Med*. 2018;25(2):205-220. https://doi.org/10.1111/acem.13296
7. McLaughlin C, Barry W, Barin E, et al. Multidisciplinary simulation-based team training for trauma resuscitation: a scoping review. *J Surg Educ*. 2019;76(6):1669-1680. https://doi.org/10.1016/j.jsurg.2019.05.002
8. Colman N, Figueroa J, McCracken C, Hebbar K. Simulation-based team training improves team performance among pediatric intensive care unit staff. *J Pediatr Intensive Care*. 2019;8(2):83-91. https://doi.org/10.1055/s-0038-1676469
9. Figueroa MI, Sepanski R, Goldberg SP, Shah S. Improving teamwork, confidence, and collaboration among members of a pediatric cardiovascular intensive care unit multidisciplinary team using simulation-based team training. *Pediatr Cardiol*. 2013;34(3):612-619. https://doi.org/10.1007/s00246-012-0506-2
10. McLaughlin CM, Wieck MM, Barin EN, et al. Impact of simulation-based training on perceived provider confidence in acute multidisciplinary pediatric trauma resuscitation. *Pediatr Surg Int*. 2018;34(12):1353-1362. https://doi.org/10.1007/s00383-018-4361-y
11. Merritt C, Overly F. An adolescent boy with systemic toxicity due to smoke inhalation. *MedEdPORTAL*. 2014;10:9863. https://doi.org/10.15766/mep_2374-8265.9863
12. Weinberger L, von Besser K. House fire victim simulation case. *MedEdPORTAL*. 2011;7:9047. https://doi.org/10.15766/mep_2374-8265.9047
13. Ventre K. A 2-year-old victim of an MVA. *MedEdPORTAL*. 2010;6:8247. https://doi.org/10.15766/mep_2374-8265.8247
14. Rando J, Bedard A, Ten Eyck R. Emergency department management of a multiple trauma patient with a left ventricular assist device. *MedEdPORTAL*. 2014;10:9984. https://doi.org/10.15766/mep_2374-8265.9984
15. Rudolph JW, Simon R, Dufresne RL, Raemer DB. There's no such thing as "nonjudgmental" debriefing: a theory and method for debriefing with good judgment. *Simul Healthc*. 2006;1(1):49-55. https://doi.org/10.1097/01266021-200600110-00006
16. Leviter JI, Sojar S, Ayala NK, Wing R. Thyrotoxicosis in a postpartum adolescent: a simulation case for emergency medicine providers. *MedEdPORTAL*. 2020;16:10967. https://doi.org/10.15766/mep_2374-8265.10967
17. Smidt A, Balandin S, Sigafoos J, Reed VA. The Kirkpatrick model: a useful tool for evaluating training outcomes. *J Intellect Dev Disabil*. 2009;34(3):266-274. https://doi.org/10.1080/13668250903093125

Received: March 18, 2023
Accepted: November 20, 2023
Published: February 27, 2024

Copyright © 2024 Vrablik and Wing. This is an open-access publication distributed under the terms of the Creative Commons Attribution license.
